# Radiation Dose-Effect Relation in Patients with Esophageal Squamous Cell Carcinoma: A National Cancer Center Data and Literature-Based Analysis

**DOI:** 10.1155/2022/2438270

**Published:** 2022-10-22

**Authors:** Weiming Han, Chen Li, Wei Deng, Wenjie Ni, Xiao Chang, Linrui Gao, Shijia Wang, Xin Wang, Zefen Xiao

**Affiliations:** ^1^Department of Radiation Oncology, National Cancer Center/National Clinical Research Center for Cancer/Cancer Hospital, Chinese Academy of Medical Sciences and Peking Union Medical College, Beijing 100021, China; ^2^Department of Radiation Oncology, Peking University School of Oncology, Beijing Cancer Hospital and Beijing Institute for Cancer Research, Beijing 100142, China; ^3^Department of Radiation Oncology, Beijing Shijitan Hospital, Capital Medical University, Ninth School of Clinical Medicine, Peking University, School of Oncology, Capital Medical University, Beijing 100038, China

## Abstract

**Introduction:**

Despite receiving definitive chemoradiotherapy (dCRT) with radiation dose (RTD) of 50.4 Gy, survival of esophageal carcinoma was dismal. The effect of RTD in cancer control and radiotoxicity, and the extent to which local-regional control (LRC) influenced survival remain vague. This study aimed at evaluating RTD-effect relationship in esophageal squamous cell carcinoma (ESCC).

**Methods:**

1440 dRT/CRT-treated ESCC patients were enrolled. Restricted cubic spline regression model was applied to reveal nonlinear relationship between RTD and survival/radiotoxicity. Linear regression analysis (LRA) was performed to evaluate correlations between LRC and overall survival (OS) or progression-free survival (PFS).

**Results:**

For 1440 dRT/CRT-treated ESCC patients, with RTD escalating, hazard ratios (HRs) of OS, PFS, LRC declined until RTD exceeded 60 Gy, then increased. HR of treatment-related mortality was stable until RTD exceeded 60 Gy, then increased. HR of LRC was lower for majority of patients treated with RTD≥60 Gy, except for those with KPS<80, T1-2 lesion, or without lymph node metastasis. LRA revealed strong correlations between LRC and OS/PFS. 45.5% and 44.9% of OS and PFS improvements were owing to improved LRC.

**Conclusions:**

RTD of 60 Gy was well tolerated, with favorable survival resulted of LRC improvement in local-advanced ESCC. Further stratification analyses based on radiation sensitivity will be helpful to determine potential beneficiaries of RTD escalation.

## 1. Introduction

With the evidence provided by the Radiation Therapy Oncology Group (RTOG) trial 85–01,^1^ concurrent chemoradiotherapy (cCRT) has become the standard treatment option for inoperable locally advanced esophageal carcinoma. Nevertheless, because of poor treatment efficacy, EC remains the 9th most common cancer and the 5th leading cause of cancer-related deaths worldwide [1]. Despite receiving the standard definitive CRT (dCRT) with a radiation dose of 50.4 Gy, 50% of the patients with EC show local-regional treatment failure with the survival time being ≤27.3 months [2]. Poor local-regional control (LRC) and overall survival (OS) remain significant problems for patients treated with dCRT. There seems to be still some room for enhancing LRC. Dose escalation is one of the potential ways of improving LRC. However, conventional radiation technique based RTOG 94–05 trial failed in improving survival or local-regional control through escalating radiation dose to 64.8 Gy [3]. However, radiation dose of 60 Gy in dCRT was acceptable in Europe and Japan and applied in clinical trials [4, 5]. Several phase 1/2 clinical trials have revealed the safety and superior survival outcomes in patients received definitive radiotherapy of radiation dose boost over 60 Gy [6–8]. But two recently published phase 3 clinical trials demonstrated survival benefit was not attained with a radiation dose over 60 Gy [9, 10]. The optimal radiation dose for definitive treatment of ESCC remains debatable in the era of conformal RT and intensity-modulated RT (IMRT). According to the studies on neoadjuvant CRT, 27% to 45% of the patients could attain complete tumor regression after receiving chemoradiotherapy with a radiation dose of 50–50.4 Gy, [11–14] in other words, there were still 55% to 73% of patients with residual cancer processing low quality of life (QoL) due to persistent of dysphagia and remaining in higher risk of disease progression. Poor LRC, OS, and QoL remain considerable problems for patients receiving dCRT with a standard radiation dose of 50–50.4 Gy [2, 3, 15, 16]. Extension of local-regional control and dysphagia relieved time span could be expected through radiation dose escalation and toxicities due to radiation dose escalation have been reported to be well tolerated [6–8, 17]. Hence, the current study aimed at exploring the radiation dose-effect relationship in dCRT with modern radiation techniques and evaluating the dose-response relationship of LRC with progression-free survival (PFS) and OS in dCRT-treated patients with ESCC through the data of a national cancer center and published literature [15].

## 2. Materials and Methods

### 2.1. Patient Population

Between January 2002 and December 2016, 2186 patients diagnosed with histopathologically confirmed EC treated with definitive RT (dRT) or dCRT were evaluated. The inclusion criteria were as follows: (1) patients with disease of stages II to IVB according to the 6th edition of the American Joint Committee on Cancer (AJCC) staging manual, (2) diagnosed with nonsquamous cell carcinoma, (3) without other malignancies within 5 years before receiving CRT, (4) without distant organ/tissue metastasis before CRT, (5) with a KPS score ≥70 and treated with dCRT, (6) without radical surgery before or after CRT, (7) underwent either 3-dimensional conformal RT, IMRT, or volumetric modulated arc therapy, and not 2-dimensional RT, (8) cumulative radiation dose, converted to equivalent dose in 2 Gy/*f* (EQD2), between 40 and 70 Gy, and (9) available at first follow-up. Eventually, 1440 patients were enrolled (Supplement Figure 1). Patients were followed up until death or April 2019. The median follow-up duration was 50.8 months, and tumor and treatment characteristics were collected. All patients had written informed consent of treatment and the Independent Ethics Committee of CAMS approved the project (no. 21/095–2766).

### 2.2. Pretreatment Evaluations and Treatment

Pretreatment evaluations included medical history-taking; physical examination; barium esophagram; computed tomography (CT) of the neck, chest ,and abdomen; and esophagogastroduodenoscopy (EGD) with endoscopic ultrasonography (EUS) and biopsy. Positron emission tomography/CT (PET/CT) was recommended but not performed as a routine examination. As the number of metastatic lymph nodes defined through clinical multimodal imagine examination was not coincided well with postoperative pathological reports. The 6th edition of the AJCC tumor-node-metastasis (TNM) staging system was applied for the exclusion of the exact number of metastatic lymph nodes. The M1 stage subgroup in the current study comprised patients with primary tumor located in upper/middle third esophagus and metastasis in celiac lymph nodes or with primary tumor located in middle/lower third esophagus and metastasis in cervical lymph nodes, without distant organ/tissue metastasis. Acute toxicities during treatment were evaluated according to Common Terminology Criteria for Adverse Events 4.03.

Patients received 3-dimension conformal RT, IMRT, or volumetric modulated arc therapy with/without cCRT. The overall radiation dose, converted to EQD2, was between 40 and 70 Gy (median: 60 Gy). To calculate the EQD2, the following formula was used: EQD2 = *D* × (*d* + *α*/*β*)/(2 Gy + *α*/*β*) [18]. *D* was defined as the prescribed total radiation dose, *d* was defined as the prescribed radiation dose per fraction, the *α*/*β*-value was a measure of the fractionation sensitivity of the cells: cells with a higher *α*/*β* are less sensitive to the sparing effect of fractionation. For the gross target volume of esophageal carcinoma, the *α*/*β*-value was 10. The radiation dose per fraction ranged between 1.8 Gy and 2.3 Gy. The gross tumor volume (GTV) was defined as visible primary tumor delineated by physicians using all possible resources (barium esophagram, CT, EGD, EUS, and if available, PET/CT), metastatic regional nodes. The clinical target volume (CTV) consisted of primary tumor plus a 0.6–0.8 cm circumferential margin, a 3 cm craniocaudal margin and metastatic regional nodes plus a 0.5 cm margin in all directions and covered the corresponding lymphatic drainage region. The planning target volume (PTV) is defined as CTV plus a uniform 0.5 cm margin. Six hundred and fifty-nine (45.8%) patients were treated with dCRT and comprised 502 (76.2%), 73 (11.1%), and 84 (12.7%) cases treated with platinum-paclitaxel, 5-fluorouracil-platinum, and other therapies, respectively.

### 2.3. Statistical Analyses

OS, PFS, LRC, distant metastasis, cancer-specific mortality (CSM), and treatment-related mortality (TRM) time were calculated from the date of initiation of RT to the date of death resulted of any cause, any evidence of progression (recurrence of the primary tumor or in the regional lymph nodes, or metastasis in distant lymph nodes or distant organs) or death, relapse of the primary tumor or in the regional lymph nodes, metastasis in other organs, death resulted of EC, death resulted of treatment-related comorbidities, or the most recent follow-up. The Kaplan–Meier method was used to estimate survival probabilities, and the log-rank test was used for statistical comparisons in patient subgroups. A Cox proportional hazard regression model was used to identify independent prognostic factors and assess the effects of radiation dose in several subgroups. The proportional hazards assumption was checked with Schoenfeld's global test before establishing the Cox regression model. All statistical tests were two-sided, and *p* < 0.05 was considered to indicate statistical significance.

Restricted cubic spline (RCS) method in the Cox proportional hazard regression model and logistics regression model allows the determination of nonlinear relationships between continuous predictors, for example, radiation dose in this study, and hazard ratios (HRs) of OS, PFS, LRC, and the results were expressed in terms of HR curves and confidence bands, considering a specific covariate value as a reference. The linear regression analysis (LRA) was applied to evaluate the relationship between LRC and OS or PFS. The LRC and survival outcome information for external validation was extracted from the literature after searching PubMed by using the following keywords, reading titles and abstracts, and confirming whether the endpoint definitions were in concordance with the corresponding definitions in this study: (“esophagus” [Title] or “esophageal” [Title] or “oesophageal” [Title]) and (“tumor” [Title] or “cancer” [Title] or “carcinoma” [Title] or “neoplasm” [Title]) and (“radiation” [Title] or “radiotherapy” [Title] or “radiotherapy” [Title] or “chemoradiation” [Title] or “chemoradiotherapy” [Title]). All statistical calculations were performed with the *R* software, version 3.6.2 (R Foundation for Statistical Computing, Vienna, Austria).

## 3. Results

### 3.1. Patient Characteristics

In the current study, 1440 patients with pathologically confirmed ESCC who were treated with dRT/CRT from 2002 through 2016 were enrolled. Most patients were male (81.8% of all cases). Almost 30% of the patients were aged ≥70 years, and 85.4% of the patients were diagnosed with disease of stages III to IV according to the 6th edition of the AJCC staging system. Most patients received RT/CRT with the radiation dose being 50–60 Gy (270, 18.8%) or 60–66 Gy (988, 68.6%). Patients' clinicopathologic characteristics are listed in Table 1. The 1-, 3-, and 5-year overall survival rates were 67.1%, 33.1%, and 24.5%, respectively, and the median survival period was 19.4 months.

### 3.2. Radiation Dose-Effect Relationship of Survival Outcomes

Both univariable and multivariable analyses indicated that sex, Karnofsky performance status (KPS), the 6th edition of the AJCC TNM staging system, tumor location, radiation dose, and concurrent chemotherapy were related to OS (Supplement Table 1 and Supplement Figure 2). In subgroup analysis, the 1-, 3-, 5-year OS were significantly different between the RT group and CRT group (64.2% vs. 70.5%, 30.3% vs. 36.5%, and 21.1% vs. 29.0%, *p* < 0.001), <60 Gy group and ≥60 Gy group (57.0% vs. 70.4%, 24.8% vs. 35.9%, and 18.0% vs. 26.7%, *p* < 0.001). Similarly, the 1-, 3-, 5-year PFS were significantly different between the RT group and CRT group (48.1% vs. 51.9%, 21.8% vs. 27.5%, and 14.6% vs. 22.8%, *p* < 0.001), <60 Gy group and ≥60 Gy group (41.1% vs.52.7%, 17.7% vs. 26.5%, and 12.1% vs. 19.8%, *p*=0.007).

**Figure 1:**
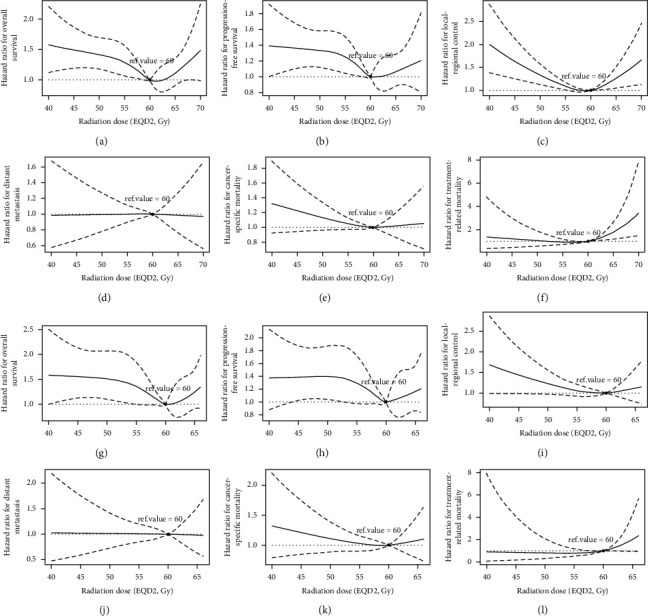
Smooth HR curves of OS (a), PFS (b), LRC (c), DM (d), CSM (e), TRM (f) for patients who received dRT/CRT and OS (g), PFS (h), LRC (i), DM (j), CSM (k), and TRM (l) for patients who received dCRT.

After adjusting for the prognostic factors mentioned above to reduce the influence of these confounders, the RCS method in the Cox proportional hazard regression model revealed nonlinear relationships between continuous radiation dose and the survival outcomes. With the radiation dose escalating, the HRs of death, disease progression, local-regional recurrence decreased until the radiation dose approached 60 Gy and increase subsequently (Figures 1(a)–1(c)), while the HR of distant metastasis was continuously stable (Figure 1(d)). The HR of death resulted of EC continuously declined till radiation dose approximately reached 60 Gy and generally remained stable afterwards (Figure 1(e)). Conversely, the trend of HR of death resulted of treatment-related morbidities was stable and subsequently enhanced as radiation dose exceeded 60 Gy (Figure 1(f)).

As the proportion of patients receiving concurrent chemotherapy was fewer in patients treated with radiation dose ≥66 Gy (Supplement table 2), the sensitivity analysis of patients treated with dCRT was applied. For 659 patients in dCRT subgroup, the trends of HRs of OS, PFS analogously declined until the radiation dose reached approximately 60 Gy and then increased (Figures 1(g) and 1(h)). The HRs of local-regional relapse and cancer-specific mortality decreased as radiation dose escalating, then remained in a steady level when radiation dose exceeded 60 Gy (Figure 1(i) and 1(k)). With the escalation of radiation dose, HR of treatment-related mortality was in a steady till radiation dose approached 60 Gy and increased afterwards (Figure 1(l)), while HR of distant metastasis remained stable (Figure 1(j)).

### 3.3. Radiation Dose-Effect Relationship of Radiotoxicities

**Figure 2:**
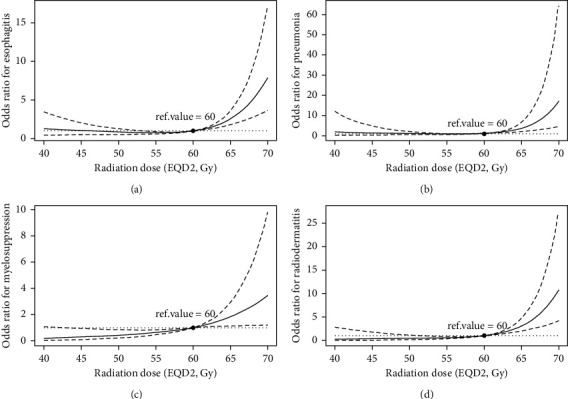
Smooth OR curves of esophagitis (a), pneumonia (b), myelosuppression (c), and radiodermatitis (d).

After adjusting for the prognostic factors of sex, KPS, the 6th edition of the AJCC TNM staging system, tumor location and concurrent chemotherapy status to reduce the influence of these confounders, the RCS method in the logistics regression model revealed nonlinear relationships between continuous radiation dose and radiotoxicities of esophagitis, pneumonia, myelosuppression, and radiodermatitis. The trends of odds ratios (ORs) of esophagitis, pneumonia, myelosuppression, and radiodermatitis were similar in a steady low level till radiation dose approached 60 Gy and then enhanced sharply (Figures 2(a)–2(d)).

### 3.4. Effects of Radiation Dose on LRC

**Figure 3:**
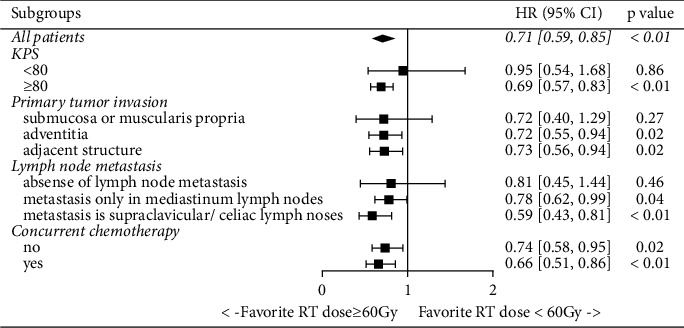
Stratification forest plots of LRC between patients treated with radiation dose ≥60 Gy and <60 Gy.

The Cox proportional hazard regression revealed a 29.3% (15.5%–40.9%, *p* < 0.01) decline in patients treated with a higher RT dose (EQD2 ≥ 60 Gy) compared to that in those treated with a lower RT dose (EQD2 < 60 Gy). In terms of subgroup analysis (Figure 3), patients having a KPS score ≥80, primary tumor invaded in adventitia or adjacent structure, lymph node metastasis in mediastinum, supraclavicular, or celiac lymph nodes, with or without concurrent chemotherapy, tended to achieve significantly better LRC with a radiation dose of ≥60 Gy. For patients with a KPS <80, primary tumor invaded in submucosa or muscularis propria, or absence of lymph node metastasis, although HRs of LRC were lower than 1 with respect to the higher radiation dose group compared to the lower radiation dose group, the differences were not statistically significant.

### 3.5. Correlation between LRC and OS/PFS

**Figure 4:**
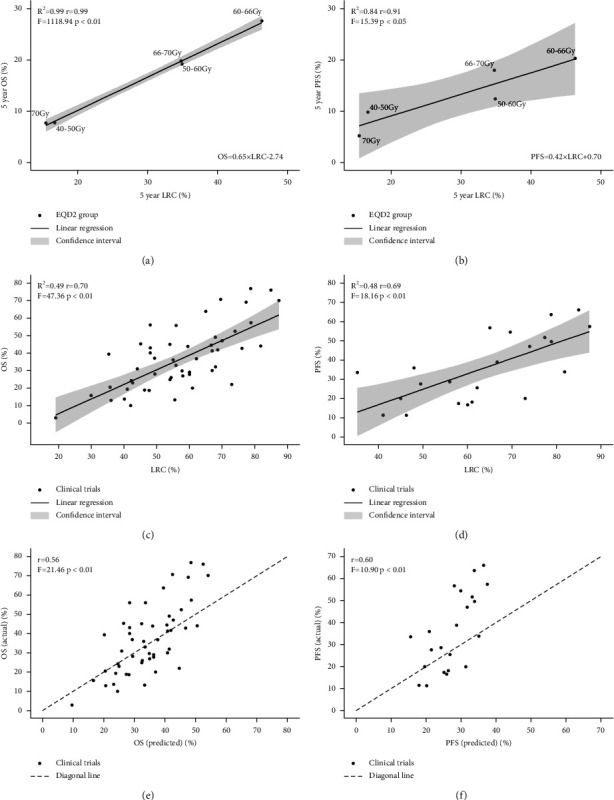
Linear regression of LRC and OS, PFS according to radiation dose (a, b), external validation data (c, d), and OS, PFS prediction efficiency of the regression model (e, f).

The 5-year OS, PFS, and LRC of patients treated with several radiation dose levels ranged from 7.7% to 27.6%, 5.3% to 20.3%, and 15.5% to 46.4%, respectively. Patients who received RT with an EQD2 range of 60–66 Gy tended to show better OS, PFS, and LRC (Supplement table 3). LRA revealed strong correlations between LRC and OS (*p* < 0.01) and LRC and PFS (*p* < 0.05). An absolute advancement of 1% in LRC could translate into an improvement of 0.65% in OS and 0.42% in PFS (Figures 4(a) and 4(b)).

OS, PFS, and LRC information from 42 studies were collected (Supplement table 4) and applied as external validation to demonstrate the relationships among these endpoints. Analogous strong correlations were confirmed between LRC and OS as well as LRC and PFS (Figures 4(c) and 4(d), *p* < 0.01); 45.5% of the OS improvement and 44.9% of the PFS improvement was attributable to enhancement in the LRC. When the linear regression formula of the current study was used to predict OS or PFS based on LRC information from the 42 studies, the predicted OS and PFS were significantly associated with the actual OS and PFS (Figures 4(e) and 4(f), *p* < 0.01 and *p* < 0.05).

## 4. Discussion

RT is an indispensable part of the intended curative approach for inoperable local advanced esophageal carcinoma [19]. After receiving standard dCRT with the guideline-preferred radiation dose of 50.4 Gy, approximately 50% of the patients showed local-regional failure in 2 years [3, 15, 16, 20, 21]. Meanwhile, 90% of local-regional failures were within the gross tumor volume (GTV) [2]. As the dominant histological subtype of EC, ESCC shows genomic characteristics more reminiscent of other SCCs [22] and presents with a higher local recurrence rate than esophageal adenocarcinoma (EAC) [16]. Thus, a higher radiation dose might be reasonable in patients with ESCC because of its potential to enhance the LRC. For reference, the recommended radiation dose for SCCs originating in other sites (e.g., head and neck, lung, thymic, and cervical SCCs) was 60 Gy or higher rather than 50 Gy. However, local control and survival benefit were not seen in RTOG 94–05 [3]. Since that study was performed from 1995 to 1999, the administration of 2-dimensional RT (2D-RT) with lower precision in dose distribution may have led to higher organ at risk (OAR) dose delivery. The subsequent excessive OAR toxicity caused a significant prolongation of treatment time because of toxicity-induced breaks in treatment. Meanwhile, seven of the 11 deaths in the high-dose arm occurred in patients who had received 50.4 Gy or less as well as a significantly lower actual dose of 5 FU as a percentage of the protocol dose. The damage caused by severe adverse events, the breaks in treatment, and inadequate treatment intensity essentially resulted in an inability to improve OS. With the implementation of IMRT, the increased dose-delivery precision can yield favorable OAR toxicity, low risks of treatment interruption, and adequate treatment intensity and thereby allow safe escalation of radiation doses to potentially obtain more satisfactory survival outcomes.

Dose-volume metrics and biological modeling of the trade-off in tumor coverage and OAR sparing in dose escalation for CRT indicated that escalation from 50 Gy to 62.5 Gy provided significant gains of more than 18% in tumor control (from 38.3% to 56.3%), with only a modest increase in predicted toxicity [23]. In terms of validation of clinical data, several prospective and retrospective clinical trials had reported improved local control and favorable treatment tolerance with radiation dose escalation. The phase 1 trial conducted by Yu et al. [6] demonstrated selective dose boost of 62.5 Gy in primary tumor and involved lymph nodes and 70 Gy in pretreatment 50% SUVmax area of the primary tumor was well tolerated, absent of any Grade 3 or higher acute toxicities causing continuous interruption of radiation for over 1 week and with favorable 1 year OS (69.2%) and LRC (77.4%). The phase 1/2 trial conducted by Chen et al. [7] revealed chemoradiotherapy with simultaneous integrated boost to primary tumor and involved lymph nodes with radiation dose up to 63 Gy could attain superior 2 year OS and LRC comparing with contemporaneous institutional cohort receiving standard dose (HR, 0.49; 95%CI, 0.26–0.92; *P*=0.03 and 0.66; 95%CI, 0.47–0.94; *P*=0.02, respectively). In the current study, Cox proportional hazard regression stratification forest plots analogously showed that improvement in LRC could be obtained with a radiation dose of 60 Gy or higher in most subgroups (patients with good performance status and locally advanced stage, with or without concurrent chemotherapy). Furthermore, the correlation between improved LRC and survival benefit had been demonstrated in several previous studies. However, the extent to which LRC influenced survival outcomes and how gains of LRC were converted to survival improvement still remain vague. The linear regression models of the survival data from our center and published clinical trials revealed a conversion relationship between LRC and survival outcomes, and nearly 50% of the improvement in OS and PFS (48% for OS and 49% for PFS) was attributable to LRC enhancement. Thus, it was rational to assume that escalation in the radiation dose could lead to LRC improvement, and the LRC improvement would subsequently convert to survival benefit. However, along with the survival benefits derived from enhancing LRC, the radiation dose-dependent toxicities emerged gradually. The nonlinear continuous dose-response smooth HR curve showed once the radiation dose exceeded 60 Gy, the risk of radiotoxicities and treatment-related mortality increased sharply. There was a threshold of survival outcomes (OS or PFS) to radiation dose, since the HR descended until the radiation dose reached 60 Gy and ascended subsequently. Escalation of the radiation dose beyond 60 Gy did not yield additional gains in OS or PFS. This could be attributed to the loss of survival benefits resulting from radiation toxicity itself and the effects of the accompanying discontinuous treatment in overwhelming the LRC-associated survival gain achieved with high-dose radiotherapy. The optimal radiation dose was deemed to balance the toxicity-related survival impairment and the tumor control-associated survival gain to the greatest extent.

In contrast to the current study and other studies supporting radiation dose escalation-associated LRC and survival gains, several previous prospective clinical trials failed to reproduce the results presented above. The ARTDECO study [8] failed in attaining 3-year OS or local-regionalprogression-free survival (LRPFS) improvement (high dose arm vs. standard-dose arm, 39% vs. 42%, *p*=0.22, and 59% vs. 52%, *p*-0.08). There was a marginal significant LRPFS improvement of 8% in the high dose arm. As the sample size of this phase 3 trial was calculated based on the hypothesis of a respectively large enhancement (15%) in local control, for reference, the LRC enhancement of the current study was 11.4% between patients treated with radiation dose of 50–60 Gy and 60–66 Gy. The hypothesis of a respectively large enhancement could lead to respectively small sample size with insufficient power to confirm the local-regional enhancement. Meanwhile, the planning target volume of the integrated boost dose merely consisted of the primary tumor but excluded the involved lymph nodes. As lymph node response to chemoradiotherapy potentially affect the treatment response of primary tumor, and 45.7% (48/105) and 51.4% (54/105) of patients with lymph nodes persistent resulted in partial and no response in the primary tumor [24], dose escalation to involve lymph nodes could be the essential way to improve the treatment response of positive regional lymph. Besides, the incidence of grade 5 adverse event was respectively high (10%) in high dose arm, partially contributed to more proportion of patients disable to complete the treatment schedule. In addition to statistical power of sample size, definition of integrated boost target volume and respectively high incidence of treatment-related mortality, the contradictory results of previous prospective and retrospective studies might be attributable to heterogeneities in the study population. Previous studies had demonstrated patients with higher clinical stage tended to have lower response rate after receiving chemoradiotherapy with standard radiation dose of 50.4 Gy [25], and survival outcomes were inferior in patients who without response to dCRT in comparison with responder [26, 27]. For patients showing a noncomplete metabolic response in positron emission tomography-computer tomography (PET-CT) scans after completing a radiation dose of 50.4 Gy, a dose escalation of 10–20 Gy could result in improved OS and LRC [20]. On the other hand, when achieving clinical complete response, patients who received CRT with a radiation dose of 45–50 Gy could achieve satisfactory 5-year OS (57.0%) and PFS (34.6%) [28]. For reference, the pCR rate of neoadjuvant CRT with a radiation dose of 40–48 Gy ranged from 27% to 45% [11–14]. The mixed study population with various radiation sensitivities may have caused uncertainty in the study conclusions. The phase 1/2 trial conducted by Welsh et al. [8] showed SIB to primary tumor and involved lymph nodes with radiation dose of 58.8–63 Gy reduced the local failure rate for patients with node-positive disease (13% vs 56%, *P*=0.04) or stage III-IV disease (29% vs 55%, *P*=0.04) comparing with patients receiving chemoradiotherapy with radiation dose of 50.4 Gy without an SIB. Analogously, subgroup analysis of the current study revealed that patients with KPS ≥ 80, indicating superior treatment tolerance, and patients with T3-4 lesion or lymph node metastasis, indicating a higher tumor burden, tended to show survival benefit in radiation dose escalation. The negative findings of recently published prospective clinical trials [9, 10] may be partially attributed to the heterogeneous nature of the study population. That study population included a large proportion of patients without lymph node metastasis (accounting for 30.4% and 26.1% of the cases). Thus, subgroups with the potential to achieve improved LRC from dose escalation should be carefully identified by more preclinical and clinical studies in the future. The optimal radiation dose may vary in patients with different radiation sensitivities, performance status, and tumor burdens. Stratification analyses based on real-world data with a large population and a radiation sensitivity prediction model based on multimodal and radiomic examinations will be helpful to determine the potential beneficiaries and design the randomized controlled trials accordingly.

The main limitation of the current study was its retrospective nature, which may have introduced some bias in the results and conclusion. Thus, the results should be validated in well-designed randomized controlled trials or retrospective studies based on real-world data with a large population. In addition, detailed information for several parameters could not be collected and enrolled in the current study, including clinical details of concurrent chemotherapy intensity, planning parameters of radiotherapy, the incidence of long-termtreatment-related toxicity, and other radiological and histopathological details.

## 5. Conclusion

Radiation dose of 60 Gy could yield favorable LRC and acceptable toxicities for patients with local advanced ESCC receiving definitive RT/CRT. The LRC gains obtained from radiation-dose escalation could convert to survival benefit to a large extent. In patients with KPS < 80, of early TNM stage and with respectively high radiation sensitivity, 50 Gy might be a sufficient definitive radiation dose. Clinicians should realize radiation-dose escalation is the potential way to improve the unfavorable LRC and survival outcomes in dRT/CRT-treated ESCC patients. The findings of the current study could serve as evidence for formulating treatment plans and designing additional prospective stratification randomized controlled trials.

## Figures and Tables

**Table 1:** Baseline characteristics.

Characteristics		No. (%)
Age	<70 years	1017 (70.63%)
	≥70 years	423 (29.37%)
	Median (range)(years)	63 (33–89)

Sex	Male	1178 (81.81%)
	Female	262 (18.19%)

KPS	<80	152 (10.56%)
	≥80	1288 (89.44%)

T Stage (AJCC 6th)	T1	32 (2.22%)
	T2	147 (10.21%)
	T3	622 (43.19%)
	T4	639 (44.38%)

N stage (AJCC 6th)	N0	214 (14.86%)
	N1	1226 (85.14%)

M stage (AJCC 6th)	M0	1046 (72.64%)
	M1a	145 (10.07%)
	M1b	249 (17.29%)

TNM stage (AJCC 6th)	Stage IIA	121 (8.40%)
	Stage IIB	90 (6.25%)
	Stage III	835 (57.99%)
	Stage IVA	145 (10.07%)
	Stage IVB	249 (17.29%)

Tumor location	Upper third	502 (34.86%)
	Middle third	689 (47.85%)
	Lower third	249 (17.29%)

Radiation technique	3D-CRT	118 (8.19%)
	IMRT	1225 (85.07%)
	VMAT	97 (6.74%)

Radiation dose (EQD2)	≥40 Gy, < 50 Gy	88 (6.11%)
	≥50 Gy, < 60 Gy	270 (18.75%)
	≥60 Gy, < 66 Gy	988 (68.61%)
	≥66 Gy, < 70 Gy	74 (5.14%)
	70 Gy	20 (1.39%)
	Median(range)(Gy)	60 (40–70)

Induction chemotherapy	No	1353 (93.96%)
	Yes	87 (6.04%)

Concurrent chemotherapy	No	781 (54.24%)
	Yes	659 (45.76%)

^*∗*^KPS = Karnofsky performance status, EQD2 = equivalent dose in 2 Gy per fraction, 3D-CRT = 3-dimensional conformal radiotherapy, IMRT = intensity-modulated radiotherapy, and VMAT = volumetric modulated arc therapy.

## Data Availability

The data underlying this article will be available from the corresponding author on reasonable request.

## Supplementary Materials

Supplementary Materials Supplement Figure 1. CONSORT diagram of patient selection. Supplement Figure 2. Forest plots of Cox proportional hazard regression multivariable analysis. Supplement Table 1. Log-rank univariable analysis of overall survival. Supplement Table 2. Proportion of concurrent chemotherapy in several radiation dose groups. Supplement Table 3. Survival outcome of radiation dose groups. Supplement table 4. Survival outcome of clinical trails.
