# Supplementary material for: Radiation Dose-Effect Relation in Patients with Esophageal Squamous Cell Carcinoma: A National Cancer Center Data and Literature-Based Analysis
Source: J Oncol. 2022 Oct 22;2022:2438270. doi: 10.1155/2022/2438270 (PMC9617729; doi:10.1155/2022/2438270)
Supplement: Supplementary Materials — Supplement Figure 1. CONSORT diagram of patient selection. Supplement Figure 2. Forest plots of Cox proportional hazard regression multivariable analysis. Supplement Table 1. Log-rank univariable analysis of overall survival. Supplement Table 2. Proportion of concurrent chemotherapy in several radiation dose groups. Supplement Table 3. Survival outcome of radiation dose groups. Supplement table 4. Survival outcome of clinical trails. [file 2438270.f1.docx]

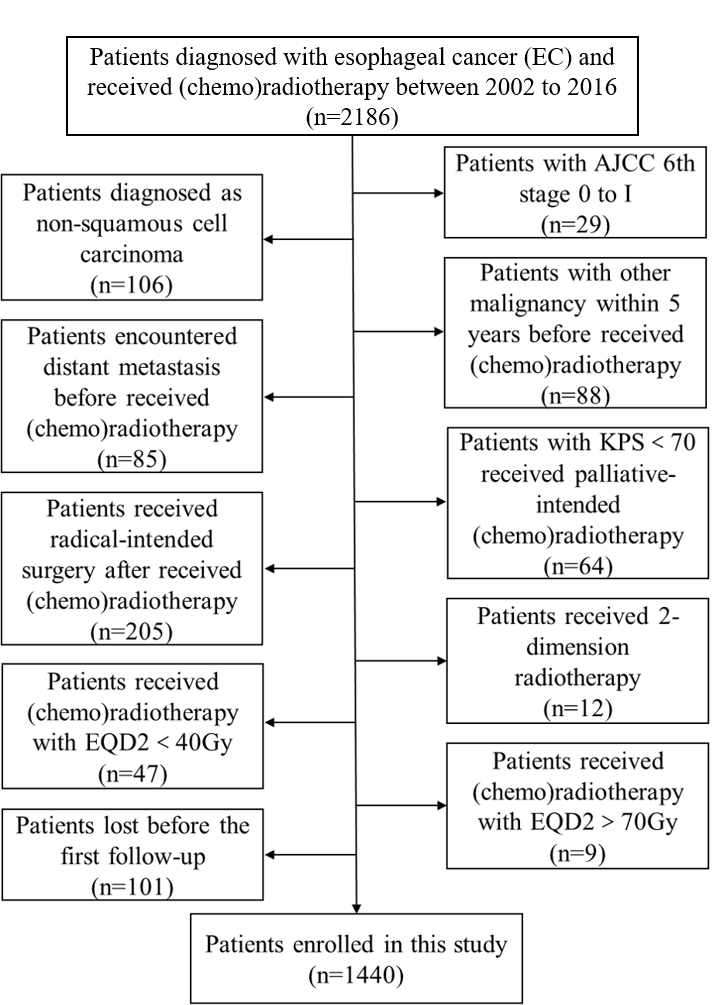


Supplement figure 1. CONSORT diagram of patient selection.


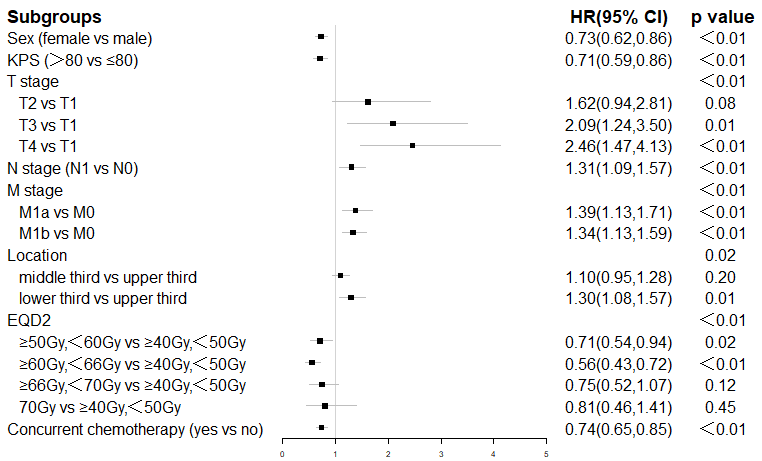


Supplement figure 2. Forest plots of Cox proportional hazard regression multivariable analysis

| Supplement table 1 log-rank univariable analysis of overall survival | | |
| --- | --- | --- |
| Characteristic | χ^2^ | p value |
| Age (＜70 years vs ≥70 years) | 1.06 | 0.30 |
| Sex (male vs female) | 19.59 | ＜0.01 |
| KPS (＜80 vs ≥80) | 14.61 | ＜0.01 |
| T stage (T1 vs T2 vs T3 vs T4) | 28.79 | ＜0.01 |
| N stage (N0 vs N1) | 10.95 | ＜0.01 |
| M stage (M0 vs M1a vs M1b) | 14.15 | ＜0.01 |
| TNM stage (stage ⅡA vs stage ⅡB vs stage Ⅲ vs stage ⅣA vs stage ⅣB) | 39.55 | ＜0.01 |
| Location (upper third vs middle third vs lower third) | 13.21 | ＜0.01 |
| Radiation Technique (CRT vs IMRT vs VMAT) | 3.86 | 0.15 |
| EQD2 (≥40Gy,＜50Gy vs ≥50Gy,＜60Gy vs ≥60Gy,＜66Gy vs ≥66Gy,＜70Gy vs 70Gy) | 37.68 | ＜0.01 |
| Induction chemotherapy (no vs yes) | 1.86 | 0.17 |
| Concurrent chemotherapy (no vs yes) | 12.70 | ＜0.01 |
| *KPS=Karnofsky performance status, EQD2=equivalent dose in 2Gy per fraction,3D-CRT=3-dimension conformal radiotherapy, IMRT=intensity modulated radiotherapy, VMAT=volumetric modulated arc therapy. | | |

| Supplement table 2 Proportion of concurrent chemotherapy in several radiation dose groups. | | |
| --- | --- | --- |
| Radiation dose | No. (%) | |
|  | Without concurrent chemotherapy | With concurrent chemotherapy |
| ≥40Gy,＜50Gy | 35 (39.8) | 53 (60.2) |
| ≥50Gy,＜60Gy | 154 (57.0) | 116 (43.0) |
| ≥60Gy,＜66Gy | 527 (53.3) | 461 (46.7) |
| ≥66Gy,＜70Gy | 46 (62.2) | 28 (37.8) |
| 70Gy | 19 (95.0) | 1 (5.0) |

| Supplement table 3 survival outcome of radiation dose groups. | | | | | | |
| --- | --- | --- | --- | --- | --- | --- |
| Radiation dose (EQD2) | 5-year OS (%) | Log-rank  p value | 5-year PFS (%) | Log-rank  p value | 5-year LRC (%) | Log-rank  p value |
| ≥40Gy,＜50Gy | 7.81 | ＜0.01 | 9.84 | ＜0.01 | 16.68 | ＜0.01 |
| ≥50Gy,＜60Gy | 19.21 |  | 12.41 |  | 34.94 |  |
| ≥60Gy,＜66Gy | 27.60 |  | 20.34 |  | 46.36 |  |
| ≥66Gy,＜70Gy | 19.80 |  | 17.99 |  | 34.79 |  |
| 70Gy | 7.70 |  | 5.28 |  | 15.48 |  |
| * EQD2=equivalent dose in 2Gy per fraction, OS=overall survival, PFS=progression-free survival, LRC=local-regional control. | | | | | | |

| Supplement table 4 survival outcome of clinical trails. | | | | | | | | | | | |
| --- | --- | --- | --- | --- | --- | --- | --- | --- | --- | --- | --- |
| Author | | year | study type | time span | No. | Stage | Treatment | RT dose | LRC(%) | OS(%) | PFS(%) |
| Anderluh et.al.^1^ | | 2019 | retrospective | 5 years | 55 | T1-T4, N0-N+ | cCRT | 57.6-70Gy | 41.00 | 19.40 | 11.50 |
| Yu et.al.^2^ | | 2015 | prospective | 1 years | 25 | Stage ⅡA-ⅣB | cCRT | 63-70Gy | 77.40 | 69.20 | 51.70 |
| Kato et.al.^3^ | | 2011 | prospective | 5 years | 76 | Stage Ⅱ-Ⅲ | cCRT | 60Gy | 62.20 | 36.80 | 25.60 |
| Waters et.al.^4^ | | 2002 | prospective | 2 years | 72 | T1-T4, N0-N+ | cCRT | 54Gy | 81.90 | 44.10 | 33.80 |
| Conroy et.al.^5^ | FOLFOX group | 2014 | prospective | 3 years | 134 | Stage ⅡA-ⅣB | cCRT | 50Gy | 61.00 | 19.90 | 18.20 |
|  | PF group |  |  |  | 133 | StageⅠ-ⅣB | cCRT | 50Gy | 58.00 | 26.90 | 17.40 |
| Zhang et.al.^6^ | | 2014 | retrospective | 3 years | 80 | T1-T4, N0-N+ | cCRT | 50-64Gy | 46.25 | 18.80 | 11.30 |
| Roeder et.al.^7^ | | 2014 | retrospective | 3 years | 27 | T1-T4, N0-N+ | cCRT | 19.2-62Gy | 48.00 | 56.00 | 36.00 |
| Chen et.al.^8^ | | 2018 | retrospective | 5 years | 812 | Stage Ⅱ-Ⅲ | cCRT | 50.4-66Gy | 49.45 | 28.10 | 27.60 |
| Kato et.al.^9^ | | 2013 | prospective | 3 years | 51 | Stage Ⅱ-Ⅲ | cCRT | 50.4Gy | 65.00 | 63.80 | 56.60 |
| Suh et.al.^10^ | HD group | 2014 | retrospective | 2 years | 77 | Stage Ⅱ-Ⅲ | cCRT | 43.2-60Gy | 74.00 | 52.40 | 47.00 |
|  | SD group |  |  |  | 49 | Stage Ⅱ-Ⅲ | cCRT | 60-75.6Gy | 45.00 | 45.20 | 20.00 |
| Lee et.al.^11^ | | 2009 | prospective | 2 years | 18 | Stage Ⅱ-Ⅲ | cCRT | 60Gy | 69.60 | 70.70 | 54.40 |
| Kawaguchi et.al.^12^ | | 2011 | retrospective | 3 years | 68 | Stage Ⅰ | RT/cCRT | 60-70Gy | 85.00 | 76.00 | 66.00 |
| Lertbutsayanukul et.al.^13^ | | 2017 | retrospective | 2 years | 44 | T2-T4, N+ | cCRT | Median 60Gy | 56.00 | 55.90 | 28.60 |
| Li et.al.^14^ | | 2010 | prospective | 2 years | 24 | Stage Ⅰ-ⅣA | cCRT+target therapy | 60Gy | 87.50 | 70.10 | 57.40 |
| Ordu et.al.^15^ | | 2015 | retrospective | 5 years | 168 | Stage Ⅰ-ⅣA | cCRT | 49-75Gy | 73.00 | 22.00 | 20.00 |
| Shim et.al.^16^ | | 2012 | prospective | 3 years | 36 | Stage Ⅲ-ⅣA | cCRT | 54Gy | 60.00 | 27.80 | 16.70 |
| Kim et.al.^17^ | | 2018 | retrospective | 2 years | 62 | T1-T4, N0-N+ | RT/cCRT | 45-90Gy | 78.90 | 57.30 | 49.60 |
| Zhai et.al.^18^ | | 2013 | prospective | 2 years | 18 | Stage Ⅱ-Ⅳ | RT+target therapy | 46-70Gy | 66.70 | 44.40 | 38.90 |
| Zhang et.al.^19^ | | 2015 | retrospective | 3 years | 102 | Stage Ⅱ-Ⅲ | cCRT | 50-70Gy | 35.30 | 39.30 | 33.60 |
| Li et.al.^20^ | | 2019^20^ | prospective | 1 years | 53 | Stage ⅡA-ⅣB | cCRT | 59.92Gy | 78.80 | 76.90 | 63.60 |
| Clavier et.al.^21^ | HD group | 2013 | retrospective | 1 years | 83 | Stage Ⅰ-ⅣA | cCRT | 50.7-72Gy | 29.80 | 15.72 | - |
|  | SD group |  |  |  | 60 | Stage Ⅰ-ⅣA | cCRT | 38-50.4Gy | 42.20 | 24.26 | - |
| Kumar et.al.^22^ | RT group | 2007 | prospective | 5 years | 60 | T1-T3, N0-N+ | RT | 36-64Gy | 40.00 | 13.70 | - |
|  | CRT group |  |  |  | 65 | T1-T3, N0-N+ | cCRT | 47-67Gy | 54.00 | 24.80 | - |
| Onozawa et.al.^23^ | | 2009 | retrospective | 3 years | 102 | Stage Ⅰ-ⅣB | cCRT | 60Gy | 48.04 | 43.00 | - |
| Button et.al.^24^ | | 2009 | retrospective | 2 years | 145 | Stage Ⅰ-ⅣA | cCRT | 50Gy | 49.30 | 37.00 | - |
| Morota et.al.^25^ | | 2009 | retrospective | 3 years | 74 | Stage Ⅰ-ⅣB | cCRT | 60Gy | 54.00 | 45.00 | - |
| Higuchi et.al.^26^ | | 2014 | prospective | 3 years | 42 | T4/M1(LNM) | cCRT | 50.4-61.2Gy | 59.52 | 43.90 | - |
| Minsky et.al.^27^ | | 1999 | prospective | 5 years | 45 | T1-T4, N0-N+ | cCRT | 64.8Gy | 61.00 | 20.00 | - |
| He et.al.^28^ | HD group | 2014 | retrospective | 5 years | 56 | Stage Ⅰ-Ⅳ | cCRT | 52.2-66Gy | 68.70 | 41.70 | - |
|  | SD group |  |  |  | 137 | Stage Ⅰ-Ⅳ | cCRT | 41.4-50.4Gy | 55.90 | 33.00 | - |
| Ohtsu et.al.^29^ | | 1999 | prospective | 3 years | 45 | T4/M1(LNM) | cCRT | 60Gy | 42.59 | 23.00 | - |
| Ishida et.al.^30^ | | 1996 | prospective | 2 years | 45 | T4/M1(LNM) | cCRT | 60Gy | 55.56 | 13.30 | - |
| Iwase et.al.^31^ | | 2013 | prospective | 5 years | 116 | Stage ⅡA-ⅣA | cCRT | 60Gy | 57.80 | 29.80 | - |
| Crosby et.al.^32^ | | 2004 | retrospective | 5 years | 90 | Stage Ⅰ-ⅣA | cCRT | 50Gy | 54.44 | 26.00 | - |
| Al-Sarraf et.al.^33^ | RT group | 1997 | prospective | 2 years | 62 | T1-T3, N0-N+ | RT | 64Gy | 42.00 | 10.00 | - |
|  | RT-CT group |  |  |  | 61 | T1-T3, N0-N+ | cCRT | 50Gy | 55.00 | 36.00 | - |
| Amini et.al.^34^ | | 2014 | retrospective | 5 years | 141 | Stage Ⅰ-ⅣB | cCRT | 50.4Gy | 68.00 | 32.00 | - |
| Ishikura et.al.^35^ | | 2003 | retrospective | 5 years | 217 | Stage Ⅰ-ⅣA | cCRT | 60Gy | 60.00 | 29.00 | - |
| Liu et.al.^36^ | IFI group | 2014 | retrospective | 3 years | 99 | Stage Ⅰ-Ⅳ | cCRT | 60-68.4Gy | 68.00 | 49.00 | - |
|  | ENI group |  |  |  | 70 | Stage Ⅰ-Ⅳ | cCRT | 60-68.4Gy | 70.00 | 47.00 |  |
| Seung et.al.^37^ | | 2004 | prospective | 3 years | 18 | Stage Ⅰ-ⅣA | cCRT | 59.4Gy | 67.00 | 30.00 |  |
| Zhang et.al.^38^ | higher dose group | 2005 | retrospective | 3 years | 26 | Stage Ⅱ-Ⅲ | cCRT | 54-64.8Gy | 36.00 | 13.00 |  |
|  | lower dose group |  |  |  | 43 | Stage Ⅱ-Ⅲ | cCRT | 30-51Gy | 19.00 | 3.00 |  |
| Ma et.al.^39^ | conventional dose group | 2017^39^ | prospective | 2 years | 60 | T2-T4, N0-N+ | cCRT | 50.4Gy | 35.70 | 20.50 |  |
|  | dose-escalation group |  |  |  | 42 | T2-T4, N0-N+ | cCRT | 60.4-70.4Gy | 76.20 | 42.80 |  |
| Wang et.al.^40^ | | 2006 | retrospective | 5 years | 35 | Stage Ⅰ-Ⅲ | cCRT | 24.5-64.8Gy | 47.70 | 18.60 |  |
| Chen et.al.^41^ | | 2019 | prospective | 2 years | 46 | Stage Ⅰ-Ⅳ | cCRT | 63Gy | 67.00 | 41.30 |  |
| Minsky et.al.^42^ | HD group | 2002 | prospective | 2 years | 109 | T1-T4, N0-N+ | cCRT | 64.8Gy | 44.00 | 31.00 |  |
|  | SD group |  |  |  | 109 | T1-T4, N0-N+ | cCRT | 50.4Gy | 48.00 | 40.00 |  |

Reference:

1 Anderluh F, Toplak M, Velenik V et al. Definitive radiochemotherapy in esophageal cancer - a single institution experience. Radiol Oncol 2019; 53 (4): 480-487.

2 Yu W, Cai XW, Liu Q et al. Safety of dose escalation by simultaneous integrated boosting radiation dose within the primary tumor guided by (18)FDG-PET/CT for esophageal cancer. Radiother Oncol 2015; 114 (2): 195-200.

3 Kato K, Muro K, Minashi K et al. Phase II study of chemoradiotherapy with 5-fluorouracil and cisplatin for Stage II-III esophageal squamous cell carcinoma: JCOG trial (JCOG 9906). Int J Radiat Oncol Biol Phys 2011; 81 (3): 684-690.

4 Waters JS, Tait D, Cunningham D et al. A multicentre phase II trial of primary chemotherapy with cisplatin and protracted venous infusion 5-fluorouracil followed by chemoradiation in patients with carcinoma of the oesophagus. Ann Oncol 2002; 13 (11): 1763-1770.

5 Conroy T, Galais M-P, Raoul J-L et al. Definitive chemoradiotherapy with FOLFOX versus fluorouracil and cisplatin in patients with oesophageal cancer (PRODIGE5/ACCORD17): final results of a randomised, phase 2/3 trial. The Lancet Oncology 2014; 15 (3): 305-314.

6 Zhang X, Li M, Meng X et al. Involved-field irradiation in definitive chemoradiotherapy for locally advanced esophageal squamous cell carcinoma. Radiat Oncol 2014; 9: 64.

7 Roeder F, Nicolay NH, Nguyen T et al. Intensity modulated radiotherapy (IMRT) with concurrent chemotherapy as definitive treatment of locally advanced esophageal cancer. Radiat Oncol 2014; 9: 191.

8 Chen Y, Guo L, Cheng X et al. With or without consolidation chemotherapy using cisplatin/5-FU after concurrent chemoradiotherapy in stage II-III squamous cell carcinoma of the esophagus: A propensity score-matched analysis. Radiother Oncol 2018; 129 (1): 154-160.

9 Kato K, Nakajima TE, Ito Y et al. Phase II study of concurrent chemoradiotherapy at the dose of 50.4 Gy with elective nodal irradiation for Stage II-III esophageal carcinoma. Jpn J Clin Oncol 2013; 43 (6): 608-615.

10 Suh YG, Lee IJ, Koom WS et al. High-dose versus standard-dose radiotherapy with concurrent chemotherapy in stages II-III esophageal cancer. Jpn J Clin Oncol 2014; 44 (6): 534-540.

11 Lee SJ, Ahn BM, Kim JG et al. Definitive chemoradiotherapy with capecitabine and cisplatin in patients with esophageal cancer: a pilot study. J Korean Med Sci 2009; 24 (1): 120-125.

12 Kawaguchi Y, Nishiyama K, Miyagi K et al. Patterns of failure associated with involved field radiotherapy in patients with clinical stage I thoracic esophageal cancer. Jpn J Clin Oncol 2011; 41 (8): 1007-1012.

13 Lertbutsayanukul C, Tharavej C, Klaikeaw N et al. High dose radiation with chemotherapy followed by salvage esophagectomy among patients with locally advanced esophageal squamous cell carcinoma. Thorac Cancer 2017; 8 (3): 219-228.

14 Li G, Hu W, Wang J et al. Phase II study of concurrent chemoradiation in combination with erlotinib for locally advanced esophageal carcinoma. Int J Radiat Oncol Biol Phys 2010; 78 (5): 1407-1412.

15 Ordu AD, Nieder C, Geinitz H et al. Radio(chemo)therapy for locally advanced squamous cell carcinoma of the esophagus: long-term outcome. Strahlenther Onkol 2015; 191 (2): 153-160.

16 Shim HJ, Kim DE, Hwang JE et al. A phase II study of concurrent chemoradiotherapy with weekly docetaxel and cisplatin in advanced oesophageal cancer. Cancer Chemother Pharmacol 2012; 70 (5): 683-690.

17 Kim JW, Kim TH, Kim JH et al. Predictors of post-treatment stenosis in cervical esophageal cancer undergoing high-dose radiotherapy. World J Gastroenterol 2018; 24 (7): 862-869.

18 Zhai Y, Hui Z, Wang J et al. Concurrent erlotinib and radiotherapy for chemoradiotherapy-intolerant esophageal squamous cell carcinoma patients: results of a pilot study. Dis Esophagus 2013; 26 (5): 503-509.

19 Zhang P, Xi M, Zhao L et al. Clinical efficacy and failure pattern in patients with cervical esophageal cancer treated with definitive chemoradiotherapy. Radiother Oncol 2015; 116 (2): 257-261.

20 Li C, Ni W, Wang X et al. A phase I/II radiation dose escalation trial using simultaneous integrated boost technique with elective nodal irradiation and concurrent chemotherapy for unresectable esophageal Cancer. Radiat Oncol 2019; 14 (1): 48.

21 Clavier JB, Antoni D, Atlani D et al. [Definitive chemoradiotherapy for esophageal cancer: 66Gy versus 50Gy, a retrospective study]. Cancer Radiother 2013; 17 (3): 221-228.

22 Kumar S, Dimri K, Khurana R et al. A randomised trial of radiotherapy compared with cisplatin chemo-radiotherapy in patients with unresectable squamous cell cancer of the esophagus. Radiother Oncol 2007; 83 (2): 139-147.

23 Onozawa M, Nihei K, Ishikura S et al. Elective nodal irradiation (ENI) in definitive chemoradiotherapy (CRT) for squamous cell carcinoma of the thoracic esophagus. Radiother Oncol 2009; 92 (2): 266-269.

24 Button MR, Morgan CA, Croydon ES et al. Study to determine adequate margins in radiotherapy planning for esophageal carcinoma by detailing patterns of recurrence after definitive chemoradiotherapy. Int J Radiat Oncol Biol Phys 2009; 73 (3): 818-823.

25 Morota M, Gomi K, Kozuka T et al. Late toxicity after definitive concurrent chemoradiotherapy for thoracic esophageal carcinoma. Int J Radiat Oncol Biol Phys 2009; 75 (1): 122-128.

26 Higuchi K, Komori S, Tanabe S et al. Definitive chemoradiation therapy with docetaxel, cisplatin, and 5-fluorouracil (DCF-R) in advanced esophageal cancer: a phase 2 trial (KDOG 0501-P2). Int J Radiat Oncol Biol Phys 2014; 89 (4): 872-879.

27 Minsky BD, Neuberg D, Kelsen DP et al. Final report of Intergroup Trial 0122 (ECOG PE-289, RTOG 90-12): Phase II trial of neoadjuvant chemotherapy plus concurrent chemotherapy and high-dose radiation for squamous cell carcinoma of the esophagus. Int J Radiat Oncol Biol Phys 1999; 43 (3): 517-523.

28 He L, Allen PK, Potter A et al. Re-evaluating the optimal radiation dose for definitive chemoradiotherapy for esophageal squamous cell carcinoma. J Thorac Oncol 2014; 9 (9): 1398-1405.

29 Ohtsu A, Boku N, Muro K et al. Definitive chemoradiotherapy for T4 and/or M1 lymph node squamous cell carcinoma of the esophagus. J Clin Oncol 1999; 17 (9): 2915-2921.

30 Ishida K, Iizuka T, Ando N et al. Phase II study of chemoradiotherapy for advanced squamous cell carcinoma of the thoracic esophagus: nine Japanese institutions trial. Jpn J Clin Oncol 1996; 26 (5): 310-315.

31 Iwase H, Shimada M, Tsuzuki T et al. Concurrent chemoradiotherapy with a novel fluoropyrimidine, S-1, and cisplatin for locally advanced esophageal cancer: long-term results of a phase II trial. Oncology 2013; 84 (6): 342-349.

32 Crosby TD, Brewster AE, Borley A et al. Definitive chemoradiation in patients with inoperable oesophageal carcinoma. Br J Cancer 2004; 90 (1): 70-75.

33 al-Sarraf M, Martz K, Herskovic A et al. Progress report of combined chemoradiotherapy versus radiotherapy alone in patients with esophageal cancer: an intergroup study. J Clin Oncol 1997; 15 (1): 277-284.

34 Amini A, Ajani J, Komaki R et al. Factors associated with local-regional failure after definitive chemoradiation for locally advanced esophageal cancer. Ann Surg Oncol 2014; 21 (1): 306-314.

35 Ishikura S, Nihei K, Ohtsu A et al. Long-term toxicity after definitive chemoradiotherapy for squamous cell carcinoma of the thoracic esophagus. J Clin Oncol 2003; 21 (14): 2697-2702.

36 Liu M, Zhao K, Chen Y et al. Evaluation of the value of ENI in radiotherapy for cervical and upper thoracic esophageal cancer: a retrospective analysis. Radiat Oncol 2014; 9: 232.

37 Seung SK, Smith JW, Molendyk J et al. Selective dose escalation of chemoradiotherapy for esophageal cancer: role of treatment intensification. Semin Oncol 2004; 31 (6 Suppl 18): 13-19.

38 Zhang Z, Liao Z, Jin J et al. Dose-response relationship in locoregional control for patients with stage II-III esophageal cancer treated with concurrent chemotherapy and radiotherapy. Int J Radiat Oncol Biol Phys 2005; 61 (3): 656-664.

39 Ma J, Wang Z, Wang C et al. Individualized Radiation Dose Escalation Based on the Decrease in Tumor FDG Uptake and Normal Tissue Constraints Improve Survival in Patients With Esophageal Carcinoma. Technol Cancer Res Treat 2017; 16 (1): 75-80.

40 Wang S, Liao Z, Chen Y et al. Esophageal cancer located at the neck and upper thorax treated with concurrent chemoradiation: a single-institution experience. J Thorac Oncol 2006; 1 (3): 252-259.

41 Chen D, Menon H, Verma V et al. Results of a Phase 1/2 Trial of Chemoradiotherapy With Simultaneous Integrated Boost of Radiotherapy Dose in Unresectable Locally Advanced Esophageal Cancer. JAMA Oncol 2019; 5 (11): 1597-1604.

42 Minsky BD, Pajak TF, Ginsberg RJ et al. INT 0123 (Radiation Therapy Oncology Group 94-05) phase III trial of combined-modality therapy for esophageal cancer: high-dose versus standard-dose radiation therapy. J Clin Oncol 2002; 20 (5): 1167-1174.
